# Supplementary figures and images for: Diving behaviour of Cuvier's beaked whales (Ziphius cavirostris) off Cape Hatteras, North Carolina
Source: R Soc Open Sci. 2019 Feb 6;6(2):181728. doi: 10.1098/rsos.181728 (PMC6408375; doi:10.1098/rsos.181728)

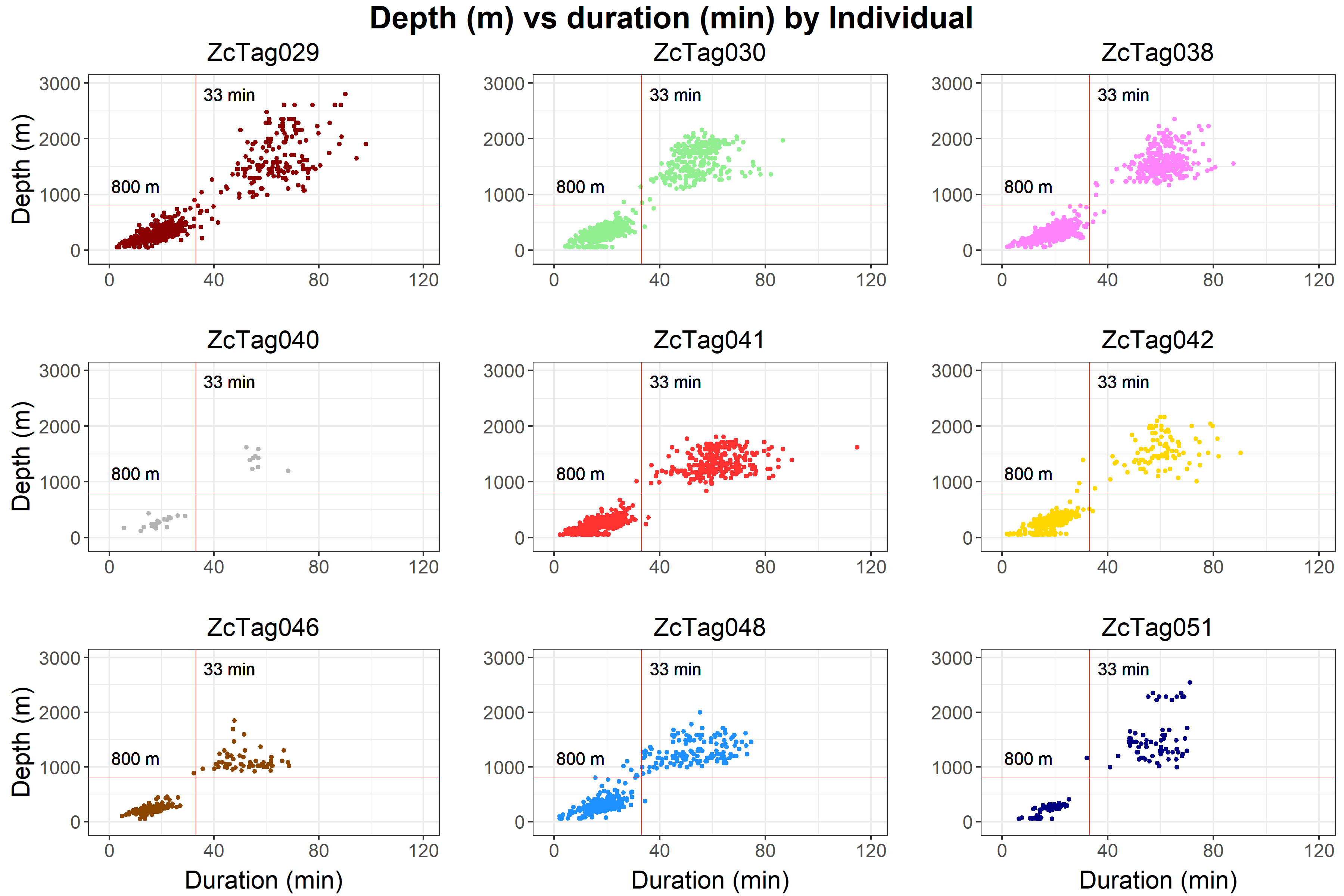

Supplement: Dive depths vs durations by individual. [file rsos181728supp1.png]
